# IM3D: A parallel Monte Carlo code for efficient simulations of primary radiation displacements and damage in 3D geometry

**DOI:** 10.1038/srep18130

**Published:** 2015-12-11

**Authors:** Yong Gang Li, Yang Yang, Michael P. Short, Ze Jun Ding, Zhi Zeng, Ju Li

**Affiliations:** 1 Key Laboratory for Materials Physics, Institute of Solid State Physics, Chinese Academy of Sciences, Hefei, 230031, China; 2 Department of Nuclear Science and Engineering, Massachusetts Institute of Technology, Cambridge, Massachusetts, 02139, USA; 3 Hefei National Laboratory for Physical Sciences at Microscale and Department of Physics, University of Science and Technology of China, Hefei, 230026, P. R. China; 4 University of Science and Technology of China, Hefei, 230026, P. R. China

## Abstract

SRIM-like codes have limitations in describing general 3D geometries, for modeling radiation displacements and damage in nanostructured materials. A universal, computationally efficient and massively parallel 3D Monte Carlo code, IM3D, has been developed with excellent parallel scaling performance. IM3D is based on fast indexing of scattering integrals and the SRIM stopping power database, and allows the user a choice of Constructive Solid Geometry (CSG) or Finite Element Triangle Mesh (FETM) method for constructing 3D shapes and microstructures. For 2D films and multilayers, IM3D perfectly reproduces SRIM results, and can be ∼10^2^ times faster in serial execution and > 10^4^ times faster using parallel computation. For 3D problems, it provides a fast approach for analyzing the spatial distributions of primary displacements and defect generation under ion irradiation. Herein we also provide a detailed discussion of our open-source collision cascade physics engine, revealing the true meaning and limitations of the “Quick Kinchin-Pease” and “Full Cascades” options. The issues of femtosecond to picosecond timescales in defining displacement versus damage, the limitation of the displacements per atom (DPA) unit in quantifying radiation damage (such as inadequacy in quantifying degree of chemical mixing), are discussed.

Radiation interaction with matter underlies radiation detectors, ion implantation and focused ion beam etching. Radiation-induced degradation of materials (e.g., void swelling, creep, hardening, embrittlement, radiation-enhanced corrosion, etc.) also presents one of the greatest obstacles to fission/fusion nuclear energy systems^1^. Innovations in nanostructured materials may yield great gains in radiation resistance^2^,^3^,^4^,^5^ due to their high sink strength originating from the large interface-to-volume ratio, as interfaces/surfaces are efficient venues for the trapping and recombination of radiation-induced mobile point/line defects^6^,^7^. Recently, many experimental and theoretical studies have been dedicated to engineering the radiation tolerance of different kinds of nanostructured nuclear materials, such as nanocrystals^8^,^9^,^10^,^11^,^12^,^13^,^14^, multilayers^15^,^16^,^17^,^18^,^19^,^20^,^21^,^22^, nanoporous solids^23^,^24^,^25^,^26^, nanowires^27^,^28^,^29^,^30^,^31^, oxide dispersion strengthened (ODS) steels^32^,^33^,^34^,^35^,^36^,^37^, nano-surface reconstruction of plasma-facing materials (PFMs)^38^,^39^,^40^ and others^41^,^42^,^43^. Understanding the radiation response of these materials requires precise knowledge of the primary radiation damage induced in their complex three-dimensional (3D) geometries.

The displacements per atom (DPA) is the most frequently applied unit to quantify radiation damage^44^,^45^. The precise definition of DPA is the following: imagine one performs molecular dynamics (MD) simulation with initial temperature *T* = 0K in the entire material. Then, one simulates the ion trajectories precisely starting with an incoming external particle, which can knock atoms out of their original “sites” if their kinetic energy right after impact (*t* = *t*_I_) exceeds *E*_d_, the displacement threshold energy, which is defined to be the largest energy barrier to overcome when displacing an atom off-site in an *ab initio* calculation. There will be heat generated, but this heat will eventually be conducted or radiated out to the surroundings, and the local temperature falls back to 0 K with no more atom movement. At this “refreezing” point *t* = *t*_F_ (which often is ~10^1^ picoseconds after the primary knock-on atom (PKA) event), one compares the refrozen configuration (that no longer evolves) with the *t* = 0 starting configuration. If the starting configuration is a perfect crystal of monatomic, isotopically pure metal, then the DPA counting exercise is simple: one just counts the number of Frenkel pairs to tally the DPA. But for nanostructures with chemical and isotopic variation, spatial gradients, and starting defect populations (interfaces, dislocation tangles, etc.), the definition of DPA is not so trivial. Indeed, consider an arbitrary compound like LiFePO_4_, trying to completely characterize radiation damage by a *single* number is a quite difficult order, since there exist a variety of damages to the Li-sublattice, Fe-sublattice, P-sublattice, and O-sublattice, with different long-term implications for material properties. Having said that, if one is to use a single number (DPA) to represent radiation damage, there are still some basic rules one should consider in the counting exercise. First, one does not distinguish between identical atoms. In other words, even though in classical MD we label atoms with numerical index, we do not distinguish between two atoms with the same atomic number (*Z*) and weight (*A*) but different index. For example, if one ^56^Fe atom exchanged with another ^56^Fe atom before and after the first-principles MD simulation, this should not count toward DPA. This is because despite the word “displacements” in displacements per atom (DPA), DPA is construed as a unit for “damage”, and permutation of two identical atoms does not count toward “damage”, despite radiation-induced primary *displacements* and heat. If one ^56^Fe atom exchanged with one ^16^O atom in a solid at *t*_F_, this should most likely count toward DPA since severe chemical damage is likely created at *t* = *t*_F_. There is also the borderline case of one ^56^Fe atom exchanging with one ^57^Fe atom before and after: while such isotopic mixing could be physically measureable (for example radiation-enhanced dissolution of an ^57^Fe inclusion in ^56^Fe matrix could influence thermal conductivity slightly), the common-sense practice is probably not to count such isotopic exchange toward DPA because not much chemical damage is left at *t*_F_.

Using *T*(*t* = 0) = 0K MD simulation gives a clean definition of DPA and other possible units of damage, but there are still two problems: (a) what is the bearing of such a definition on reality where *T*(*t*) > 0K at *t* = 0 and beyond, and (b) how to estimate DPA efficiently with SRIM-like Monte Carlo computation (with “Quick Kinchin-Pease” or “Full Cascades” options^44^,^45^), as first-principles MD simulations are expensive. For (a), the usual notion is that thermal activation is important for long-term defect evolution after *t* > *t*_F_, but not so much before, so one can take the 0 K MD simulated configuration at *t* = *t*_F_ as the initial condition for subsequent evolution, and so the defined DPA should still be a good quantification of damage at *t* = *t*_F_, due to separation of timescales. This argument becomes tricky for liquids, very-high-temperature solids (e.g. superionic materials with for example “molten” Li- or O-sublattice), and glasses, but we will not delve deeper into (a) now. For (b), achieving a reasonably good estimate for radiation damage including DPA at *t* = *t*_F_ with much lower computational cost than MD is indeed the central goal of this paper. The short answer is that the “Quick Kinchin-Pease” (QKP) option should give *faster and more accurate* DPA estimation in SRIM-like codes, including IM3D^44^. The “Full Cascades” (FC) option is much slower and also tends to give the “wrong” answer^44^, because FC is meant to give the radiation *displacement* distribution at *t* = *t*_I_ (~fs), not the *damage* distribution at *t* = *t*_F_ (~10^1 ^ps). From *t*_I_ to *t*_F_ the atomic configuration can still evolve by athermal dynamic recombination/transient annealing effects, that is, if a displaced interstitial atom is not located beyond the spontaneous recombination radius, then the Frenkel pair can still dynamically recombine even at 0 K, or during the transient thermal spike before refreezing. Thus, if one were to take one FC *displacement-off-site* event to mean one *damage* event seen at *t* = *t*_F_ (which would be wrong), SRIM FC would provide an overestimated “DPA” by a factor of around two compared to the QKP option and MD benchmark^44^. For most users of SRIM-like codes whose intent is to get damage/DPA *numbers* close to the first-principles MD definition, the “Quick Kinchin-Pease” (QKP) option is much preferred. The FC option is like an “open heart surgery” option for experienced developers who would like to understand more about the difference between radiation displacements and damage, and the limitations of a single value DPA in quantifying damage. In principle, it should be possible to evolve FC *displacements* distribution at *t* = *t*_I_ (~fs) to QKP-like damage distribution at *t* = *t*_F_ (~10^1 ^ps), taking effects like athermal recombination and transient annealing into account approximately with reasonable fidelity. Unfortunately, at present the physics engine for such *t*_I_*-*to-*t*_F_ evolution is not well developed at all, so presenting the FC-option result in any form should be taken with extreme caution.

## IM3D Overview

Ion irradiation has been widely applied to study high-DPA radiation effects in structural materials^46^, including changes in material microstructure, local properties, dose rate effects, retention of H/He atoms, surface erosion in PFMs^47^, etc. All these radiation effects in crystals originate with the direct, primary production of atomic-scale point defects, consisting of vacancies, interstitial atoms, anti-site defects, and clusters. The accumulation and migration of these atomic defects directly results in the degradation of key material properties by numerous mechanisms, such as amorphization, phase and blister formation, void swelling, irradiation-induced creep, and radiation enhanced corrosion. In comparison to bulk materials, the irradiation of nanostructures often results in far less damage accumulation, because the size of a single, ballistic collision cascade (10^−9^–10^−8 ^m) is comparable to the size of the nano-features^48^. For a small sample size in one or more dimensions like nanowires or 2D materials, only a small part of the projectile energy is deposited in the object, while both the sputtering yield and defect annealing are enhanced by the high surface-to-volume ratio^7^. Therefore, new computational implementations and a careful re-examination of the underlying physics are duly required for the modeling of the radiation effects in 3D nanostructured materials.

Despite the fact that the commonly used unit of radiation damage (DPA) is defined by first-principles MD simulation, Monte Carlo (MC) codes are very frequently used to simulate radiation damage, due to their high computational efficiency, accounting of electronic energy loss and multiple- and plural-scattering, and no limitations in nanostructure size, ion energy, or availability of empirical interatomic potentials^7^,^49^,^50^,^51^. Mayer *et al.* pointed out that the agreement between MC and MD is quite good for angular and energy distributions for the multiple small angle scattering of MeV ions^50^. MC methods are generally accurate enough to estimate the amount of ion deposition and primary radiation damage, at least in the validated ion energy range of the binary collision approximation and for ion fluences that do not produce severe amorphization, *if* the distinction between “Quick Kinchin-Pease” (QKP option for damage) and “Full Cascades” (FC option for modeling fs-scale off-lattice-site displacements) option is made, as noted in the Introduction section.

The accuracy of MC simulation codes depends critically on the accuracy of the underlying models and databases of radiation stopping power, as well as the specific implementation algorithms. SRIM (Stopping and Range of Ions in Matter) has been the *de facto* standard for stopping power calculations for more than 30 years, including single elements with atomic numbers from 1–92 and energy ranges of 10 eV-2 GeV/amu^45^. Paul also pointed out that SRIM and MSTAR (a program that can predict stopping powers for ions from ^3^Li to ^18^Ar) describe experimental data best over a wide range of targets and energies in general, by comparing recent stopping power tables for different kinds of ions with experimental data^52^,^53^. Since 2003, a self-contained SRIM module “SRModule.exe” has been published with SRIM^54^, which allows for the easy integration of SRIM’s stopping power database into external programs, thus promoting the development of new ion-matter interaction codes.

Computational efficiency is the *raison d'être* as well as major performance metric of the MC approach, which specifically occurs in the calculation of the classical binary atomic scattering angle (*θ*_CM_) expressed as an integral in the center-of-mass coordinate system^51^. SRIM-like codes use the MAGIC algorithm to compute *θ*_CM_ considering a screened Coulomb potential, which is already an improvement by at least two orders of magnitude over the direct computation of the scattering integral while sacrificing little accuracy^45^. Database interpolation has been improved by Yuan *et al.*^55^ and Schiettekatte^56^, by a fast indexing technique of exploiting the binary representation of floating-point numbers to index tables (including scattering angle components and even stopping power) in log-scale without explicitly computing log-function. These techniques exhibit a significant speed gain by several orders of magnitude compared to the MAGIC algorithm with nearly no loss of accuracy^49^,^56^.

SRIM/TRIM^45^ has been a serial instruction code, and only applies to thin films and multilayers^7^. Geometrically complex targets were usually simulated as a finite stack of two-dimensional, compositionally homogeneous material layers^7^. This works approximately as long as the size of a radiation cascade (10^−9^–10^−8 ^m) is much smaller than the size of the microstructure, but clearly breaks down for nanostructures whose geometrical sizes are comparable to that of a single cascade. Other codes have treated limited 3D cases^49^,^51^,^57^,^58^, but their computational power needs to be enhanced to meet the demands of complex 3D geometries with many domains and interfaces, and parallelization is needed. IM3D is the first parallelized MC code for general 3D structures, and demonstrates good parallel scaling performance.

In the past a simple 3D framework based on the construction of equal-sized rectangular cells has been introduced^49^,^59^. However, this type of 3D geometric model has limitations. For example, the stepped planes of the cellular geometry can distort the sputtering yield, causing atoms to re-enter the target and create further sputtering/defects. There is no correction of the step change in electronic stopping power between the cell boundaries. In addition, there are domain size adaptivity and scaling issues^49^,^59^.

Thus, it is necessary to develop a more general and robust approach in constructing arbitrarily complex 3D nanostructures. The flexibility of the geometry description “language” and the corresponding optimization of the ion tracing procedure within the material structure are certainly the two most important aspects of a 3D model. In past years, we have successfully implemented the Constructive Solid Geometry (CSG)^60^,^61^ and Finite Element Triangle Mesh (FETM) computational geometry libraries^62^,^63^ to efficiently simulate particle transport in arbitrary 3D geometries^64^,^65^. In this paper, we introduce IM3D (Irradiated Microstructures in 3D), which uses the fast database indexing technique^56^ on the scattering angle and standard physical parameters such as electronic stopping powers generated using “SRModule.exe”^54^, with the user’s choice of CSG/FETM library as the 3D structural description^64^,^65^. The code is implemented with the Message Passing Interface (MPI) library, with excellent parallel scaling performance on Beowulf clusters (up to 10^5^ times faster than serial-instruction SRIM executable). We have also compared IM3D results with SRIM in both QKP and FC options for 1D benchmark cases, and have demonstrated very close numerical agreement. An added benefit is that the collision physics engine of IM3D is open source^66^,^67^ and can be modified at will.

In the following, the framework of IM3D will be first described, followed by a number of illustrative examples of using IM3D to simulate geometrically complex nanostructures. Both “Quick Kinchin-Pease” (QKP option for damage) and “Full Cascades” (FC option for modeling *fs*-timescale off-lattice-site displacements) options are described in detail.

### IM3D Program Description

IM3D is a massively parallel, open-source, 3D MC code for simulating the transport of ions and the production of defects within materials. It can model arbitrarily complex 3D targets made of different geometric elements, each composed of different materials. Both the 3D distribution of ions and the material evolution associated with the ion’s energy loss, such as displacement, sputtering, damage, ionization, and phonon production, can be modeled by IM3D with local rules. The FC option in IM3D allows one to follow all target atom cascades in detail, and the open-source platform of IM3D allows one to modify the detailed local rules. Output parameters include electronic and nuclear energy deposition, back-scattering/implanted ions, radiation damage in DPA, point defect concentrations, and sputtered atoms, etc. Output files are in the format of .*cfg*, .*msh* or .*vtk*, which can be viewed by software such as AtomEye^68^, Gmsh^69^ and ParaView^70^.

IM3D consists of three major components: 1) physical models of ion-atom collisions, 2) universal 3D structural models, and 3) efficient computational algorithms.

#### Physical models

**Figure 1:**
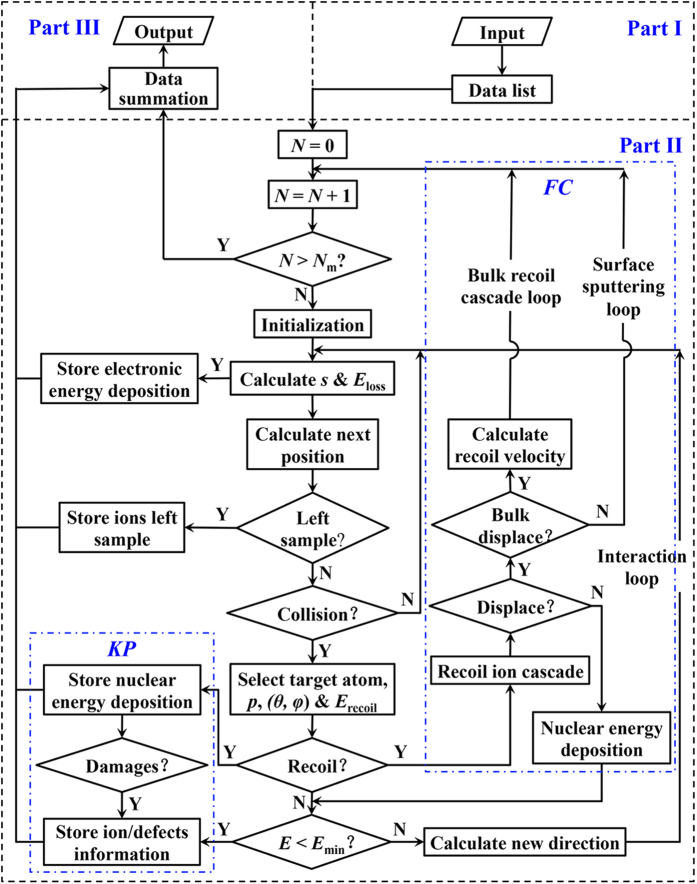
IM3D program flowchart showing ion tracking and defect generation methods.

IM3D’s collision physics are similar to well-established SRIM-like codes, which employ the random phase approximation (RPA), the binary collision approximation (BCA) and the central potential approximation (CPA)^45^,^56^,^71^. The code computes random trajectories of ions to present statistically meaningful data. Each trajectory corresponds to a particle (ion or knocked target atom) with a specified starting position, a given direction, and an incident or primary energy. The particle is tracked as a random sequence of straight free-flight-paths, ending in a binary nuclear collision event where the particle changes its direction of movement and/or loses energy as a result of nuclear (elastic) and electronic (inelastic) interactions. The energy and direction of the particle are then updated by conserving energy and momentum. The probability of energy loss depends on the target atom density, as well as the nuclear and electronic stopping powers that can be assumed to be density-independent. Meanwhile, point defects can be produced in elastic collision events. Finally, the trajectory is terminated once the energy of the particle drops below a specified value *E*_min_, or if the particle leaves the domain. A flowchart describing how each incident particle is tracked is given in Fig. 1, while a more detailed physical background can be found in ref. 45.

For the elastic collision process, the classical binary atomic scattering angle *θ*_CM_ in the center-of-mass (CM) coordinate system can be evaluated from the scattering integral as,


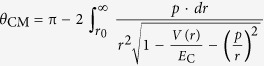


where *p* is the impact parameter (the perpendicular distance between the path of a projectile and the center of the field created by an object that the projectile is approach), *r*_0_ is the closest distance (*r*) between two nuclei during the collision, *V*(*r*) is the screened interatomic potential as listed below, and *E*_C_ is the kinetic energy of the incident atom in the CM frame. This integral cannot be calculated analytically for general interatomic screening potentials, and hence a time-consuming numerical integration is performed^45^. An analytical approximation formula (such as the MAGIC approximation^45^) or a lookup table method (such as the fast indexing technique^56^) can be used to improve the efficiency, as described in Section “Numerical algorithm and computational efficiency”. The interaction potential *V*(*r*) between two atoms is a screened, repulsive Coulomb potential described by a dimensionless screening function, such as the Thomas-Fermi potential^72^, the Lenz-Jensen potential^73^, the Moliere potential^74^, the Bohr potential^75^ or the Ziegler-Biersack-Littmark (ZBL) potential^45^. In addition, the recoil energy of a target atom due to elastic nuclear collisions can be evaluated by BCA between two charged particles involved in one scattering process.

For the inelastic collision process, the ion energy decreases uniformly along the free-flight-paths through electronic energy losses, accounting for energy straggling. In IM3D, physical parameters such as electronic stopping powers are generated using “SRModule.exe” provided by SRIM^54^. These are provided in the form of pre-calculated tables as implemented in Corteo^56^. Either the Bohr^76^, Chu^77^, or Yang^78^ formulas can be selected to account for energy straggling. Furthermore, IM3D also employs a linear mixing of stopping powers (known as Bragg’s rule^79^) for determining the total stopping power in alloys and compounds.

The displacements and generation of point defects can be modeled by the analytically modified Kinchin-Pease (KP) model^80^,^81^ or a full cascade (FC) simulation, as shown in Fig. 1. In order to improve calculation efficiency, the QKP option only traces the incoming ion trajectory and simplifies the defect generation processes accordingly, without tracking the sub-cascade processes of displaced target atoms in detail. After each ion elastic scattering event by a target atom, the Norgett-Robinson-Torrens (NRT) empirical formula^81^ is employed to estimate the total displacements/defect production in subsequent cascade processes approximately, depending on the kinetic energy acquired by the target atom (*E*_v_), as


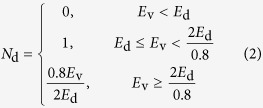


where *E*_d_ is the displacement threshold energy. Only the cumulative number of defects along ion trajectories can be given by the QKP method, and more detailed spatial information (like the exact positions of defects and the spatial correlation between them) is lost. The athermal dynamic recombination/transient annealing effects are meant to be included in Eq. (2), thus the QKP method provides the average amount of DPA/interstitials/vacancies at the refreezing time *t* = *t*_F_ (~10^1 ^ps), which can be directly compared to the 0 K MD results in most cases^1^. However, the amount of interstitials and vacancies is assumed to be equal, which is always set equal to the DPA value estimated by Eq. (2). There is also no special treatment of anti-site and chemical mixing in the QKP option. Indeed, the usage of Eq. (2) is well validated in monatomic crystals, but probably not in chemically complex crystals like LiFePO_4_.

In contrast, the FC option traces the entire cascade trajectories of both energetic ions and the displaced target atoms, and describes the displacement/defect generation pattern at *t* = *t*_I_ (~fs) in greater detail. In the FC option, a trajectory ion/atom with atomic number *Z*_1_ and energy *E* collides with a target atom with atomic number *Z*_2_. After the collision event, the energy of the trajectory ion/atom changes to *E*_1_ and the target atom obtains energy *E*_2_. Different events could then occur according to the following local rules: (1) if *E*_2_ > *E*_d_ (target atom), the target atom has acquired enough energy to leave the site. As it leaves, an energy loss *E*_L_ is subtracted from *E*_2_, and then sub-cascade will be traced. *E*_L_ is a tunable parameter in IM3D FC, with the default *E*_L_ value taken to be the surface or bulk binding energy (*E*_b_) as in SRIM FC, although there is no rigorous physical justification for this. (2) if both *E*_1_ > *E*_d_ and *E*_2_ > *E*_d_, a vacancy is created; (3) if *E*_2_ < *E*_d_, the target atom remain in its site, releasing energy *E*_2_ as phonons; (4) if *E*_1_ < *E*_r_, *E*_2_ > *E*_d_, a replacement occurs with energy *E*_1_ released as phonons. If *Z*_1_≠*Z*_2_, an anti-site will be created; otherwise no chemical damage should occur locally. The default value for the replacement threshold *E*_r_ is *E*_b_, again the lattice/surface binding energy of the target atom. However, there is no rigorous physical basis for this, and *E*_r_ can be changed to other values in IM3D that can depend on *Z*_1_ and *Z*_2_. (5) if *E*_1_ < *E*_min_ and *E*_2_ < *E*_d_, the trajectory ion/atom becomes localized here as an interstitial atom. These local rules can be modified at will by the user in open-source IM3D. The default treatment in IM3D is listed above, in order to reach numerical agreement with SRIM.

Thus, the FC option provides not only total numbers of different defects (i.e. interstitials, vacancies and anti-sites of different varieties, which satisfy sum rules with the total off-site displacements but are generally unequal with each other), but also their initial positions at *t* = *t*_I_ (~fs). In FC, the displacement tally is meant for *t* = *t*_I_ (~fs), and the exact athermal recombination/transient annealing effects of point defects from *t*_I_ to *t*_F_ is still untreated at present. After the FC run, a new physics engine (yet undeveloped) for *t*_I_*-*to-*t*_F_ evolution may reduce the number of defects to an average value comparable to that of QKP at *t*_F_, by introducing a parameterized recombination cutoff radius and a stochastic algorithm to utilize the spatial information at *t* = *t*_I_. These chemically and physically complex processes (which should require more *ab initio* energy parameters than just *E*_d_) are beyond the scope of this paper and could be pursued in future studies, which is expected to give more realistic characterization of radiation-induced chemical mixing (consider the LiFePO_4_ example) beyond single-valued DPA as characterization of radiation damage. At present, however, using the FC total displacement count at *t* = *t*_I_ (which characterizes radiation-induced *displacement*) as damage or DPA is ill-advised and strongly discouraged, as we will show below in our FC/QKP comparison.

#### 3D geometric models

**Figure 2:**
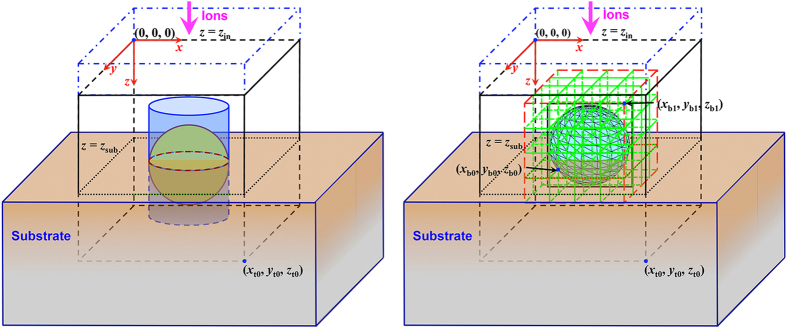
CSG (left) and FETM (right) geometric models and their corresponding coordinate systems.

3D structures can be described by two options in IM3D, the CSG and FETM methods^64^,^65^, with corresponding coordinate systems shown in Fig. 2. Ion beams with different atomic numbers and incident directions can be sampled from different spatial distributions (i.e., specified point, center point, uniform, or Gaussian random distributions). The targets are composed of different geometric elements, each containing different materials (pure elements, alloys, and compounds).

The CSG method^60^,^61^ uses simpler geometries to build a complex structure with Boolean operations: union, difference and intersection. By CSG modeling, a complex geometric structure can be constructed from a few basic, simple 3D body elements analytically described with few parameters. The basic elements, such as the sphere, tetrahedron, cuboid, ellipsoid, taper, column, polyhedron, paraboloid, hyperboloid, and others, are implemented in the code to allow efficient calculation of intersecting points of an ion trajectory with object surface. Detailed construction processes can be found in refs 60 and 61. There are two limitations. First, it is obvious that with the limited number of elementary objects, it could be difficult in practice to build certain complex geometric structures. Fortunately, many simple nanostructures (such as nanowire arrays) can be accurately modeled by CSG. Second, for the tracing of ion/atom trajectories, one must compute the intersection points with every possible basic shape. A large number of such decisions in a MC simulation would incur a heavy computational cost. An efficient parallel algorithm is required for the simulation of very complex targets constructed with CSG.

On the other hand, the FETM method^62^,^63^ allows for easier construction of an arbitrarily complex geometric structure with smooth or rough surface. Structures can be easily generated by different software, such as Gmsh^69^, or even the user’s own definition. The accepted formats of FETM shape files are *opengl* and .*ply2* (Polygon File Format) at present. The reason for using a triangular mesh is the easier determination of intersection points of a straight line with a local triangular plane, when considering an ion incident to or an atom leaving a surface. This method is more scalable for simulating very complex targets. Mesh adaptivity by domain decomposition is also implemented in the FETM framework.

In IM3D, the free-flight-paths of an ion between two successive collision events follow either a Poisson distribution with a mean free-flight-path 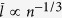 (where *n* is the atomic density of the target) or a deterministic value specified by the user. Afterwards, a ray-tracing technique^60^,^61^ for a complex geometric structure and space subdivision are introduced to accelerate the calculations^62^,^63^. Furthermore, three physical quantities, the free-flight-path, the direction deflection, and the kinetic energy, change after ion refraction at each surface/interface. These must be treated appropriately, especially at the boundaries of complex geometries^62^.

#### Numerical algorithm and computational efficiency

For the sampling of *θ*_CM_, IM3D utilizes routines from Corteo^56^ to efficiently sample the scattering and azimuthal angles and the stopping powers that are based on both the fast indexing technique and the MAGIC approximation^45^ alternatively, with improved accuracy, efficiency, and memory usage. For the calculation of the stopping power, the same algorithm for treating *θ*_CM_ can also be used by generating tables of stopping power values first, and then interpolating from them. For a small number of chemical elements, the memory burden is no more than several megabytes. A detailed treatment of the fast indexing techniques can be found in refs 49 and 56.

Based on the above acceleration techniques, a typical simulation of 10^5^ trajectories with energies of keV to MeV consumes only seconds to minutes on a 3 GHz serial computer even for complex 3D structures, and would be even faster when using the multi-threaded or parallel version of IM3D.

## Results and Discussions

### Validation and Verification

To verify the accuracy of IM3D, three example calculations regarding the ion/damage distributions in bulk and nanostructured targets are performed and compared with those of SRIM and MD. Furthermore, some issues with using SRIM-like codes are identified and clarified.

**Figure 3:**
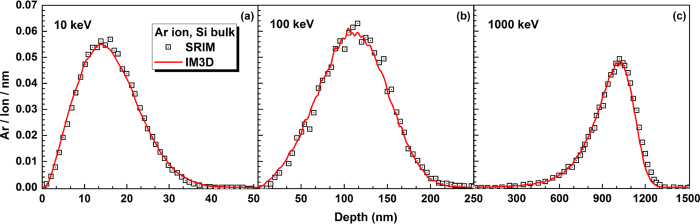
Comparison of IM3D results with SRIM predictions for Ar ion depth-distributions in Si bulk, under a total of 10^5^ Ar ions implantation with different energies of (a) 10 keV, (b) 100 keV and (c) 1000 keV and normal incidence at the center point of the target.

As shown in Fig. 3, the depth distributions of ion deposition are calculated for bulk Si under Ar ion irradiation with different irradiation energies. Profiles of ion depth-distribution are obtained, which are in very close agreement with those of SRIM calculation at all three ion energies, but much faster computationally.

**Figure 4:**
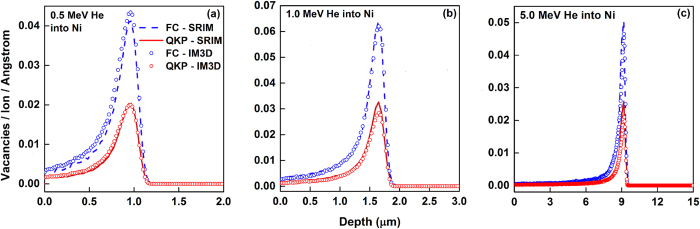
Comparison of IM3D results with SRIM predictions for vacancy depth-distributions predicted by FC and QKP methods in bulk Ni, under irradiation by 10^5^ He^+^ ions with different energies of (a) 0.5 MeV, (b) 1.0 MeV and (c) 5.0 MeV and normal incidence at the center point of the target.

The vacancy distributions for bulk Ni under He ion irradiation with different incident energies are given in Fig. 4. Again, nearly perfect agreements between IM3D and SRIM in both QKP and FC runs are demonstrated for all three ion energies. The intensity ratio between the predictions of the full cascade (FC) and Quick Kinchin-Pease (QKP) methods is roughly two. By comparing to standard reference values estimated by MD and NRT model, Stoller *et al.* attributed this discrepancy between FC and QKP to a probable fundamental problem in the SRIM FC approach used to calculate the number of vacancies^44^. Since SRIM is not open-source, the details of its displacement/defect generation algorithms are not publicly accessible in its manual or publications^45^. Because we have reproduced the SRIM results almost perfectly, it should be true that the SRIM FC values are significantly larger than QKP values because the FC option describes the off-site displacement pattern right after impact, at *t* = *t*_I_ (~fs), while the QKP option estimates the damage at the refreezing point *t* = *t*_F_ (~10^1 ^ps). This interpretational discrepancy is clearly quite fundamental, as noted in the Introduction and Program Description sections. Also, SRIM FC assumes *E*_L_ = *E*_b_ which is probably an underestimate of the local energy dissipation. In addition, Borschel *et al.* pointed out an obvious discrepancy between Iradina and SRIM for damage production within collision cascades^49^. This discrepancy should be due to the difference of the replacement fractions in the total displacement events, determined by a certain energy cut-off^49^, usually assumed to be *E*_d_ as declared in SRIM’s manual and elsewhere^45^,^49^. In fact, however, the replacement process should occur only when the following two rules are satisfied: the displacement of a target atom must first occur by obtaining enough energy (at least *E*_d_) from the trajectory atom; and the residual energy of the trajectory atom is lower than a threshold energy *E*_r_. By taking *E*_r_ = *E*_b_, we indeed reproduced simiar depth profiles as SRIM FC, with nearly identical absolute values.

In practice, the QKP/NRT method^80^,^81^ is usually considered the better estimation of the defect numbers at *t* = *t*_F_, which can compare well with MD results in most cases^44^ for chemically simple crystals. At present QKP is likely the more accurate, computationally efficient and thus more practical way to estimate the total value of primary damage in chemically simple materials, which is recommended for non-developers. The FC method can be selected to reveal the inner workings of radiation damage and generate a more fine-grained picture of the radiation displacement pattern at *t* = *t*_I_ (~fs), which are the natural initial conditions for the *t*_I_*-*to-*t*_F_ evolver (still not well-developed to date), whose outcome (spatial distributions of interstitials, vacancies and anti-sites of different varieties) can then feed into subsequent longer-term simulations like kinetic Monte Carlo (KMC) and cluster dynamics (CD)^82^. Even though today’s IM3D FC option/*t*_I_*-*to-*t*_F_ evolver can be considered “work in progress” for chemically complex materials, the open-source nature of IM3D and its nearly perfect capture of SRIM FC results with the default ruleset will facilitate this highly meaningful development in the future.

**Figure 5:**
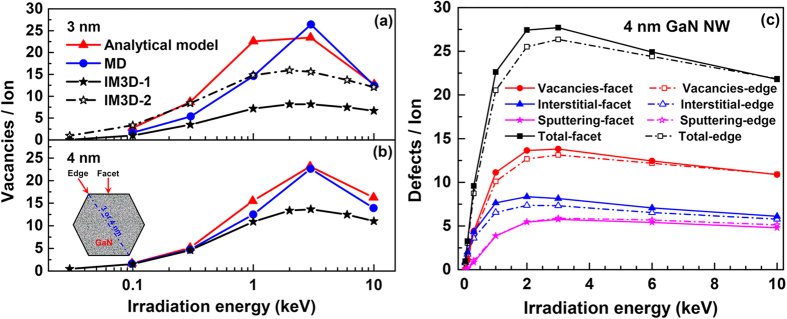
Comparison of the number of vacancies in a (a) 3 nm and (b) 4 nm-diameter GaN nanowire along with Ar ion energies predicted by IM3D, the analytical model based on SRIM, and MD simulations. The curves for the analytical model are artificially scaled to have the same area as those from MD simulations, since the absolute amount of damage cannot be estimated reliably from the results based on SRIM calculations^30^. IM3D-1 and IM3D-2 correspond to the bulk energy thresholds and half values of the bulk thresholds used in the simulations, respectively. (**c**) Separate and total accumulation of three different types of defects (vacancies, interstitials and sputtered atoms) along with Ar ion energies, for the 3 nm and 4 nm-diameter GaN nanowire under Ar ion irradiation at the edge and face of a GaN nanowire.

As the size of the nano-features becomes comparable to the sizes of the collision cascade or sub-cascades, the high surface-to-volume ratio of nanostructured materials could induce two new effects, termed the nano-energetics effect and nano-geometric effect. Due to quantum confinement, surface stress and elastic image interactions, fundamental material energetics such as electronic stopping power and *E*_d_ could change with the size reduction, which causes the nano-energetics effect. Meanwhile, the nano-geometric effect will also influence the trajectory of an ion when it moves through different material zones, in processes such as trajectory emission, re-entering, sputtering, and shading^30^. In Fig. 5(a,b), the amount of defects along with irradiation energies calculated by IM3D exhibits a similar trend to that of the analytical model and MD simulation^30^ (with the maximum amount produced at energies around 3 keV), while the absolute magnitude of damage is smaller than that from MD simulations. This discrepancy should come from the nano-energetics effect, including the difference of stopping powers and the displacement and binding energy thresholds between nanostructures and bulk. IM3D would therefore underestimate the total number of defects for both 3 and 4 nm nanowires, if the bulk energetics were used for determining the defect generation. We can reduce this discrepancy by adjusting the threshold energies, for these energy thresholds are usually lower with the size reduction of nanostructures and follow a universal relation as predicted for the cohesive energy of nanoparticles as in ref. 83. This idea was tested by using the half values of bulk energy thresholds to decrease the discrepancy between IM3D and MD simulations for the 3 nm nanowire.

Both the nano-energetics effect and nano-geometric effect can contribute to the energy-dependent discrepancies (Δ*N*_NRT_) between IM3D calculations and MD simulations for nanostructured materials. Due to the nano-energetics effect, the displacement energy *E*_d_ of nanostructured materials would decrease to 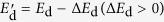, and Δ*N*_NRT_ can be simply estimated by the NRT model^81^ 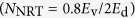, for *E*_v_ ≥ 2*E*_d_/0.8) as


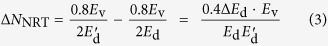


For the nano-geometric effect, the energy that goes into nuclear collisions (*E*_v_) is proportional to the total energy deposition (or the effective interaction volume) in the nanostructured material. The kinetic energy deposited to nuclei in the nanostructure, *E*_v_, increases at first with increasing irradiation energy, and then decreases after a critical energy (such as ~3 keV here) when the deposited energy starts to decrease due to ions escaping from the surface. Accordingly, with increasing irradiation energy, Δ*N*_NRT_ is dominated at first by the nano-energetics effect (a constant ratio (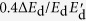) relative to *E*_v_ when the size of a nanowire is fixed), and then by the nano-geometric effect (i.e., ions escaping), as shown in Fig. 5(a,b).

In IM3D, we can consider the bulk energetic parameters to be valid when the target size is larger than *L*_C_. *L*_C_ has a value of ~20 nm, as it is known that the thermodynamic properties change less sensitively with object size above 20 nm^83^. For objects smaller than *L*_C_, IM3D can use a set of modified parameters to account for the nano-energetics effects. In addition, Fig. 5(c) shows that both the nano-energetics and nano-geometric effects determine the number of defects, demonstrating that the amount of different types of defects for ions incoming from the flat facet is higher than from an edge where two facets meet. The difference between the flat facet and edge cases is smaller than that of MD results^30^, because no crystallographic channeling effect is implemented in IM3D yet.

### Benchmark of serial and parallel efficiency

As shown in Table 1, the single CPU core computational time of IM3D, SRIM^45^ and Iradina^49^ are given to showcase the high efficiency of IM3D serial version compared to the other MC codes, for a 305 nm ZrO_2_ film on Si under a total of 10^5^ incoming Au ions with energy 2.0 MeV. In general, for 1D problems, IM3D in serial mode is faster than SRIM by at least two to three orders of magnitude, and a little faster than Iradina with the same indexing technique due to the fewer surfaces/interfaces between different domains in the simulation system.

**Figure 6:**
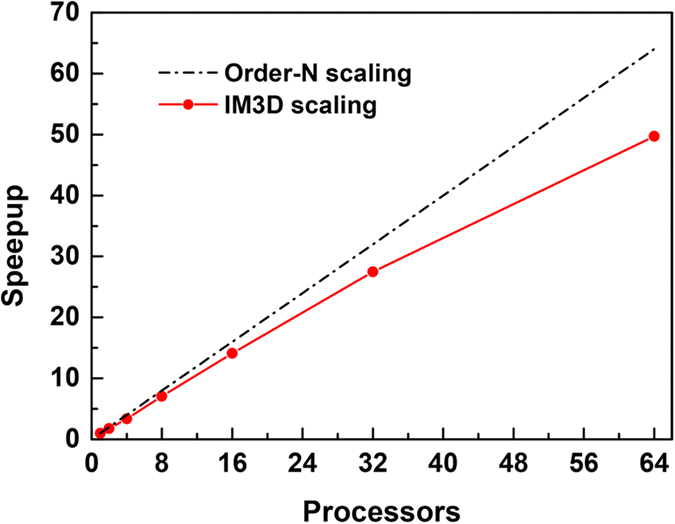
Wall clock scaling of IM3D with multi-processors running Message Passing Interface (MPI), for the system of a 305 nm ZrO_2_ film on Si under a total of 10^5^ Au ion irradiation with ion energies of 2.0 MeV.

In addition, the parallel speedup ratio of the parallel version of IM3D was tested on a Beowulf cluster, for the system of a 305 nm ZrO_2_ film on Si under a total of 10^5^ Au ions irradiation with ion energies of 2.0 MeV, as shown in Fig. 6. A nearly linear speedup ratio is achieved, with the acceleration efficiency of up to 80% up to hundreds of cores. The widespread availability of Beowulf clusters and the excellent parallel scaling performance of IM3D should make it very attractive to users and developers.

### Applications. Complex targets based on CSG and FETM methods

**Figure 7:**
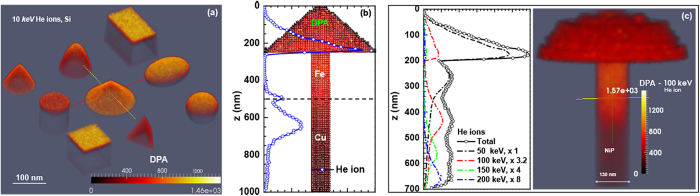
(**a**) DPA spatial distributions of nine basic shapes of pure Si constructed using the CSG method and under 10 keV He ion irradiation with a normal-incidence beam. (**b**) DPA spatial distribution in a 100 nm diameter Cu/Fe nano-bicrystal made by CSG under 200 keV He ion irradiation with a normal-incidence beam. The blue curve corresponds to the He ion depth distribution. (**c**) Separate and total He ion depth distributions for different energies He ions (left) and DPA (right) spatial distribution for 100 keV He ions with a normal-incidence beam irradiated on a 130 nm NiP metallic glass nanostructure constructed by FETM.

Nine basic shapes (in Fig. 7(a)) and their assemblies (in Fig. 7(b)) were constructed using CSG to model many regular targets with different materials. The nano-geometric effect is responsible for changes in the defect spatial distributions between different shapes (without taking into account the nano-energetics effect, which only becomes significant when the characteristics size scale drops to less than *L*_C_  =  20 nm^83^).

It can be seen in Fig. 7(b,c) that for the geometrically complex nanostructures in refs 28 and 29, errors would be introduced when using a non-3D code. As shown in Fig. 7(b), a more realistic DPA spatial distribution is obtained for a Cu/Fe nanobicrystal built with CSG, which accounts for the sputtered atoms and primary knock-on atoms leaving the material properly. For the Ni_73_P_27_ nanostructure in Fig. 7(c), in order to generate a roughly uniform DPA distribution using a combination of for implantations with energies 50, 100, 150, 200 keV, IM3D FETM calculations suggest using He ion fluences of ratio 1 : 3.2 : 4 : 8, instead of the 3.5 : 4.0 : 2.8 : 5.5 recipe in ref. 28. Thus, the nano-geometric effect can dramatically change the damage behavior of ion irradiation and cannot be neglected.

### Nano-yttria in ODS steels under ion-irradiation

ODS steels with yttria (Y_2_O_3_) nanoparticles have been shown to be a new class of radiation resistant, high-strength nuclear materials^2^,^32^,^33^,^34^,^35^,^36^,^37^. The radiation resistance mechanisms associated with the embedded nanoparticles are still somewhat controversial. It is of interest to understand localized primary radiation damage inside and around the small Y_2_O_3_ nanoparticles. Here, we assume a pure iron matrix in which Y_2_O_3_ nanoparticles are embedded as a simplified model of an ODS steel, and simulate the spatial distributions of primary radiation damage with *E*_d_ of 40 eV^45^ for Fe and 57 eV for Y and O^37^.

**Figure 8:**
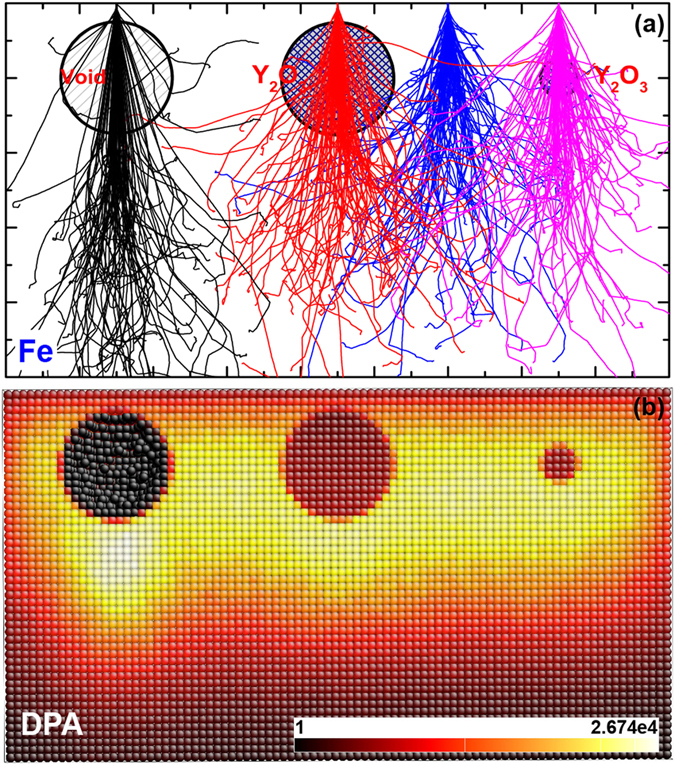
The (a) ion trajectories and (b) DPA distribution cross-section for pure iron with a void (30 nm) and two Y_2_O_3_ nanoparticles (30 nm and 10 nm) of different sizes under 150 keV Fe ion irradiation with a normal-incidence beam.

IM3D predictions of the ion trajectories and 3D spatial distribution of DPA in Fe matrix with one void and two Y_2_O_3_ nanoparticles of different sizes are shown in Fig. 8. Spheres with different materials can change ion trajectories (Fig. 8(a)) by influencing the energy losses and the production rate of defects, and finally induce the different damage distributions (in Fig. 8(b)). Compared to the bulk, ions near voids penetrate more deeply. Similarly, ions also lose less energy and generate less damage in Y_2_O_3_ nanoparticles. Thus, Y_2_O_3_ nanoparticles play a somewhatsimilar role as voids to partly suppress the localized production of primary radiation damage, but without the loss in strength that voids would induce. This is attributed to the fact that Y_2_O_3_ nanoparticles have lower atomic density (6.68 × 10^22 ^atoms/cm^3^) compared to that of the steel matrix (8.388 × 10^22 ^atoms/cm^3^), and higher displacement energies of Y and O (57 eV^37^) compared to that of Fe (40 eV^45^). The minor enhancement of DPA intensity behind the void and the larger Y_2_O_3_ nanoparticles was also found in MD^36^, which originates from the overlapping DPAs generated by the penetrating ions though the void/nanoparticle and the ions directly transporting in the iron matrix nearby, as shown in Fig. 8(a). This minor enhancement effect can be removed by decreasing the diameter of Y_2_O_3_ nanoparticles from 30 nm to 10 nm as shown in Fig. 8(b).

With lower primary defect generation rate inside and around embedded Y_2_O_3_ nanoparticles (smaller than 10 nm), and with proliferation of internal phase boundaries that could act as defect sinks and pinning sites of grain boundaries, the nano-oxides should be more stable, as verified by high-resolution TEM measurements^37^.

### Ion beam sputtering induced bending of W nanowire

**Figure 9:**
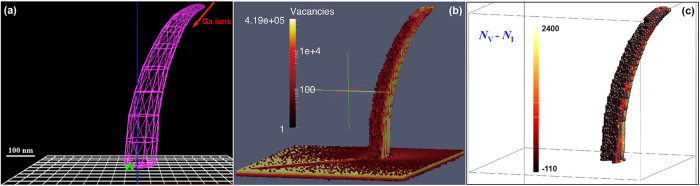
The (a) 3D surface mesh with FETM geometric method, (b) vacancy spatial distribution and (c) remaining excess vacancies for a bent W nanowire under randomly distributed Ga ion sputtering with an incident direction of 40 degrees and an energy of 150 keV.

Nanowires have been observed to bend towards ion beams under high fluences^84^, finally align with them. A tungsten nanowire structure was modeled using FETM (Fig. 9(a)), and its defect distribution and excess vacancy distributions were predicted using the FC option in IM3D (Fig. 9(b,c)) with a Ga-ion beam at a 40 degree angle from the lower surface normal. More primary vacancies were generated on the side of the nanowire facing the ion beam, locally inducing a higher compressive strain due to the negative relaxation volume of each vacancy, thus inducing a bending moment towards the ion beam direction. At finite temperatures, interstitials with a low migration energy of 0.054 eV^85^ would quickly anneal with vacancies or diffuse towards and plate at a free surface. The vacancies, meanwhile, are far less mobile with a migration energy of 1.7 eV^85^. Here, in order to consider the final remaining damage in W nanowire, we use the difference between ballistically produced vacancies and interstitials by assuming that annihilation of defects only occurs in each cell (10 × 10 × 10 nm^3^). A distribution of the excess vacancies is given in Fig. 9(c), which are more concentrated on the side towards the ion beam. Thus, a bending moment (due to residual stress from an inhomogeneous distribution of vacancies/interstitials and their relaxation volumes) towards the ion beam should be induced, until the nanowire is aligned with the ion beam, as observed in the experiment^84^. Moreover, the shading/shadowing effect (shading of the ions on their incident path by a particle leads to a decrease of damage behind the particle) can also be seen in Fig. 9(b) with dark area in the substrate appearing behind the nanowire. During plasma-surface interactions in PFMs^86^, these two effects (i.e., the bending and shading effects) should also occur at the reconstructed surface, promoting the formation of “fuzz” nanostructures on the surface.

## Summary

IM3D, an open-source massively parallel 3D Monte Carlo code for computing primary radiation *displacements* and *damage* has been developed based on explicit local rules, more scalable 3D structural models, and more efficient sampling algorithms. It is capable of investigating the 3D spatial distributions of primary radiation defects in nanostructured materials. Verification was performed with SRIM and MD, and almost exact agreement with SRIM in both “Quick Kinchin-Pease” (QKP) and “Full Cascades” (FC) modes are obtained, using a “default” set of local rules in IM3D, although these local rules can be easily modified and added. The FC result is thus construed to model the radiation *displacement* pattern at *t* = *t*_I_ (~ fs), while the QKP result is construed to measure radiation *damage* (DPA) at *t* = *t*_F_ (~10^1 ^ps). At present QKP is likely the more accurate, more efficient and more practical way to estimate the DPA distribution, which is recommended for non-developers. The FC method can be selected to reveal the inner workings of radiation damage and generate a more fine-grained picture of the radiation displacement pattern at *t* = *t*_I_ (~ fs), which are the natural initial conditions for the *t*_I_*-*to-*t*_F_ evolver (still not well-developed to date), whose outcome (spatial distributions of interstitials, vacancies and anti-sites of different varieties) can then feed into subsequent longer-term simulations like kinetic Monte Carlo (KMC). Even though today’s IM3D FC option/*t*_I_*-*to-*t*_F_ evolver can be considered “work in progress” for chemically complex materials, the open-source nature of IM3D and its capture of SRIM FC results with the default ruleset will facilitate such highly meaningful development in the future. Several examples are given to demonstrate the calculation of primary radiation damage in arbitrarily complex materials. New 3D geometry-dependent effects, such as the nano-energetics effect, the nano-geometric effect, the shading effect, etc. are demonstrated using specific examples.

IM3D is an open-source code accessible under the GNU general public license^87^, except for the parts of CSG/FETM models whose copyrights belong to Prof. Ze Jun Ding. Thus, in order to preserve these copyrights, the routines related to the CSG/FETM models are compiled as static libraries. The source code of IM3D (C language version), and all examples in this paper can be found on publicly available websites at the Institute of Solid State Physics, Chinese Academy of Sciences (ISSP, CAS), Massachusetts Institute of Technology (MIT)^66^ and our Github repository^67^.

## Additional Information

**How to cite this article**: Li, Y.G. *et al.* IM3D: A parallel Monte Carlo code for efficient simulations of primary radiation displacements and damage in 3D geometry. *Sci. Rep.* **5**, 18130; doi: 10.1038/srep18130 (2015).

## Figures and Tables

**Table 1:** Single CPU computational time of IM3D, SRIM (only serial version available)^45^ and Iradina (only serial version available)^49^ codes, for a 305 nm ZrO_2_ film on Si under 10^5^ Au ion irradiation with ion energies of 2.0 MeV.

Method	SRIM (serial only)	Iradina (serial only)	IM3D
**FC (sec)**	473269	12089 (slow), 6345 (fast)	5760
**KP (sec)**	11758	—	59
